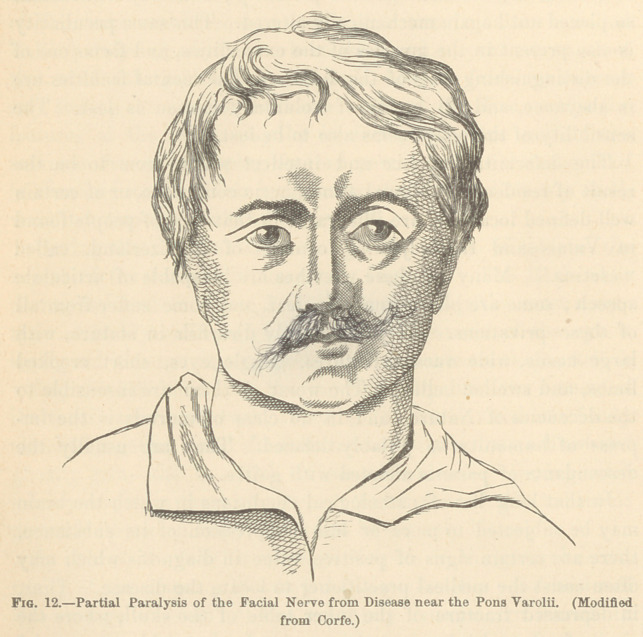# The Human Face; Its Modifications in Health and Disease, and Its Value as a Guide in Diagnosis

**Published:** 1881-01

**Authors:** Ambrose L. Ranney

**Affiliations:** Adjunct Professor of Anatomy in the Medical Department of the University of the City of New York


### Selections.

THE HUMAN FACE; ITS MODIFICATIONS IN HEALTH AND DISEASE, AND ITS VALUE AS A GUIDE IN DIAGNOSIS. By AMBROSE L. RANNEY, M.D., Adjunct Professor of Anatomy in the Medical Department of the University of the City of New York.

The extent to which the anatomy of the head, as studied from the stand-point of physiognomy, may suggest points of practical value to the physician or surgeon, has not, in my opinion, received sufficient consideration in the popular text-books of the day. From the *British and Foreign Medical Review* of 1841 I quote the following sentence: "Medical physiognomy is, in many instances, a source of diagnosis which seldom fails the practitioner who is himself well versed in it; and we believe that much of the exquisite tact in discrimination of disease, which distinguishes some practitioners and which others can never attain, depends upon the vivid perception of an eye and ear habitually familiar with the lineaments, the tone, and the gestures of disease." Among the earlier authors, who were ignorant of many of the present methods of determining the condition, size, and position of organs. since the art of auscultation and percussion is a growth of later date, the study of the human countenance formed a very important part of the preparatory drill. The followers of Hippocrates and Galen were rendered perfect in their perceptive faculties. The former gave to us, in his masterly work, descriptions of the symptoms of disease which are still considered classic, while the latter, in his essays on the "Temperaments,"* is equally careful to note the most trivial alteration either of the face or of the posture.

* Kuhn's edition.

There seems to be a growing tendency of late to regard the rational symptoms of disease as subordinate to the results of a physical examination, and as of but little value in themselves, except as confirmatory evidence. Authors frequently render the description of the symptoms of disease so terse and indefinite, that but few of the readers of the later medical or surgical works could precisely picture to themselves the *appearance* of a sufferer from any of the maladies, with the pathology and physical symptoms of which they may be thoroughly familiar. It is not infrequently the experience of the most erudite of the profession to be amazed at the gift, which is possessed by some less scholarly brother, of making a diagnosis, which seldom errs, without the aid of the thermometer or the stethoscope; and many an old nurse, long accustomed to spend weary nights in watching the sick, can often render a prognosis which seems little short of inspiration when her utter ignorance of all medical knowledge is considered.

Despite the fact that some of our best authors have denounced the attempts of De Salle, Jadelot, and Seibert to establish certain facial lines and wrinkles as of positive value in diagnosis, and have pronounced all such statements as a mere fantasy, still no one of large experience can deny that the face may at times afford most positive and valuable information.

In 1806, Lavater* published his work upon this subject, in which he discusses at great length the diagnostic value of general physiognomy. Subsequently, Sir Charles Bell wrote upon the subject from a purely anatomical point of view, and, in 1824, published his "Essays upon Expression." Baumgaertner† added his contribution to the subject in 1839, and Laycock,‡ in 1862, published his course of lectures, with illustrations, which were designed to show the various types of diathesis and their bearing upon the general development. Corfe, in 1867, published a series of contributions in the *Medical Times and Gazette*, in which the subject was studied from a clinical point

* "L'Art de connaître les Hommes par Physiognomie." Paris, 1806–'7.

† "Atlas," 1839.

‡ "Med. Times and Gaz.," 1862, vol. i.

of view, and in which not only the entire field of facial expression, but also that of general physiognomy, was pointed out to the student, so far as the cases under consideration illustrated any points of special interest.

Darwin's great work upon the expression of the emotions in animals and the contributions of Connelly * upon the typical shades of expression peculiar to the insane may well be read by those who question the utility of this much-neglected department of science. The careful study of the expressions of the face and the modifications which age produces in it, is at least very advantageous in furnishing a normal standard by which deviations in disease may be studied. I quote from the most excellent treatise of Blandin † the following sentence: "Those who neglect or seek to ridicule this mode of investigation prove only one thing, that they study pathology without a proper knowledge of anatomy and physiology, upon which the former is founded. The morbid expressions of the face are an extremely useful and often the only guide of the medical practitioner in the case of a very young child, that can tell nothing in regard to its sufferings."

It is with a view to systematize and arrange the collected investigations of the authors previously named, and to bring within the compass of a single article such practical information as the anatomy of the face may afford the practitioner, that I am led to draw professional attention to this subject once more.

The physiognomy of the sick presents innumerable shades of expression. It may assume the various conditions expressive of sadness, dejection, attentiveness, indifference, uneasiness, or terror; it may, at times, be smiling; occasionally menacing or wandering; and may sometimes show a series of changes in rapid succession.

These various conditions of the countenance may not only be the direct result of the influence of the ever-varying passions upon the muscles of the face, as is the case in health, but they may also be classed as morbid phenomena, each of which possesses some special significance. Chomel lays great stress upon

* "Med. Times and Gaz.," 1862.

† "Anatomie Topographique."

these variations of countenance, and endeavors to point out the special diagnostic value of each.

FACIAL LINES AND WRINKLES.—The theories of De Salle, Jadelot, and Seibert * as to the diagnostic value of facial lines and wrinkles have their share of support from time to time; while they have also been considered by some authors as speculative and destitute of any value. The existence of these marks may be attributable to one of two conditions, viz., a disappearance of the fat from the subcutaneous tissues of the face, or the

FIG. 1.—The Transverse Rugæ.

abnormal contraction of certain facial muscles, dependent upon some apparent irritation of the motor nerves supplying the affected muscles. It is important, in using these lines and wrinkles as guides in diagnosis, that the discrimination be made between those lines which are natural to the face of the sufferer and those which are developed as a result of the disease. For the reason that the face of the adult is always more or less marked by lines,† it must be evident that these lines are a more

* Williams, "Principles of Medicine."

† Blandin, op. cit.

reliable guide in the infant than in later life, if their diagnostic value remains unquestioned. Without entering into a discussion as to the merits of the question, I give the theories advanced for whatever interest and value they may possess to the reader. The wrinkles of the face may be classified into six groups as follows:

(1.) *The Transverse Rugæ.*—These are situated upon the forehead, and are formed by the action of the occipito-frontalis muscle. They are thought to be expressive of an extreme amount of pain, arising from causes outside of the cavities of the body.

FIG. 2.—The Oculo-frontal Rugæ.

(2.) *The Oculo-frontal Rugæ.*—These extend vertically from the forehead to the root of the nose, and are formed by the corrugator supercillii muscles. They are thought to express distress, anxiety, anguish, and *excessive pain from some internal cause.* It is said that they furthermore indicate an imperfect or false crisis; and that, in attacks of acute diseases, an impending efflorescence and sometimes a fatal termination may be indicated by their occurrence. In those types of headache where the pain is very excessive, these rugæ may exist simultaneously with the ones previously described. It is stated that when the former rugæ meet the latter abruptly, during the course of an acute disease, some serious lesion of the brain, or its coverings, is developing.

FIG. 3.—The Line of Jadelot.

(3.) *The Linea Oculo-zygomatica.*—This line (the line of Jadelot) extends from the inner angle of the eye downward and outward, passing across the face below the malar bone. It is said to indicate, in children, a *cerebral or nervous affection*;* and, in adult life, some disease of the genital organs, masturbation, or venereal excess.

* Vogel, "On Diseases of Children." New York: D. Appleton & Co.

(4.) *The Linea Nasalis* (Line of De Salle.)—This line extends from the upper border of the ala nasi downward, in a direction more or less curved, to the outer edge of the orbicularis muscle. This line is said to be strongly marked in phthisis and in atrophy. Its upper half (the linea nasalis proper) is thought to be a reliable indication of *intestinal disease*, if extensively developed and prominent; the lower half (the linea buccalis) is supposed to indicate the existence of some *disease affecting the stomach.* It is claimed by Peiper that, when this line appears conjointly with the line of Jadelot, it may be regarded as a positive indication of worms in children, if a peculiar fixed condition of the eye exists and a pallor of the face is present.

FIG. 4.—The Line of De Salle.

(5.) *The Linea Labialis.*—This line extends downward from the angle of the mouth till it becomes lost in the lower portion of the face. It is usually developed in connection with those *diseases which render breathing laborious* or *painful*, and is more common in children than in the adult as a sign of diagnostic value.

(6.) *The Linea Collateralis Nasi.*—This line extends from the nose downward to the chin, in a semicirclar direction. It lies outside of the linea buccalis, the linea nasalis, and the linea

FIG 5.—The Linea Collateralis Nasi.

labialis. It is thought to be a reliable guide to diseases of the *thoracic* and *abdominal viscera.**

COLOR OF THE FACE.—The color of the face is subject to variations which to the eye of the medical adviser afford unquestioned aid in diagnosis. *Flushing* of the face, as evidenced by a diffused redness which is of a transient character, is very common in women suffering from irregularity of the menstrual periods and during the menopause. In plethora, especially after exertion or excitement, an unnatural redness of the face may

* Corfe, "Medical Times and Gazette," 1867.

occur, associated with symptoms indicative of cerebral hyperæmia. Pressure of tumors, either of the neck, or of the thorax, upon the sympathetic nerve may create an abnormal dilatation of the capillaries, thus resulting in a redness of the skin, with an increase of the temperature of the affected region; while section of the sympathetic nerve, although a rare form of accident, would result in a like condition.* *Red patches* occur on the cheek during an attack of croupous pneumonia. In wasting affections of a chronic character, especially of the lungs, such as phthisis, cancer, etc., a circumscribed redness over the malar bones, known as the "hectic flush," is usually present. It may occasionally affect only one cheek,† where only one lung is diseased. *Pallor* of the face is the rule during convalescence from any severe disease, and in patients long deprived of sunlight.‡ A *waxy pallor* exists in chronic Bright's disease, which renders the skin almost transparent. In the chill of fevers and malarial attacks, a *dusky paleness* is usually perceived; while in cases of hæmorrhage, where the loss of blood has been sufficient to produce constitutional effects, the pallor of the face assumes a peculiar *leaden color*.§ A *greenish tint* is present in profound attacks of anæmia and during chlorosis, || giving to the face an appearance similar to that of imperfectly bleached wax.

Malaria and cancer are often manifested by a *light straw color* of the face, although it may occasionally result in the deep yellow of jaundice.¶ In the early stages of jaundice, the sclerotic coat of the eye and the corners of the mouth first show the *yellow color*, although the discoloration soon tends to become diffused over the entire face. A *blue tinge* exists in those cases where the venous return to the right heart is obstructed, or where, from any cause, the oxygenation of the blood is imperfectly performed. It occurs therefore in cynosis, asphyxia, the fevers, certain diseases of the pulmonary organs which interfere with the

---

* M. Foster.

† Stillé.

‡ Williams, op. cit.

§ Sir Charles Bell, "Treatise on Surgery."

|| Niemeyer, "Text-Book of Practical Medicine." New York: D. Appleton & Co.

¶ Reynolds. "System of Medicine."

circulation, and in diseases of the heart which render its action weak or imperfect. In cases of poisoning from the nitrate of silver, the skin assumes a still deeper blue tint than in those cases above mentioned, and the staining is permanent. In Addison's disease of the supra-renal capsules a *dark-brown color* of the skin results, which may be either uniform or in isolated spots, and which may, in severe cases, almost rival the pigmentation of the negro. The redness of erysipelas is usually accompanied by an œdema which renders the face tense and shining, and which often causes a markedly altered expression of the countenance.

The face is the seat of many of the eruptions, some of which are confined almost exclusively to it, while others are usually found in that region before they appear elsewhere. It would exceed the limits of this article to enter into the description of the characters which stamp each of the various eruptions, since they can be easily learned by reference to any of the special treatises.

Corfe suggests as a guide to the student in physiognomy the following table, which designates the prevailing changes in the complexion of the face in the course of the more common disorders. While it is not possible to construct any table which shall give all the information desired upon so important a subject, still this one may prove of some value as a means of aiding the memory:

| | | | |
|---|---|---|---|
| In cerebral disease............ | the countenance | is | lethargic. |
| In emphysema ................ | " | " | livid. |
| In pulmonary œdema......... | " | " | dusky and distressed. |
| In pneumonia ................ | " | " | dusky and flushed. |
| In pleurisy ... ....... ...... | " | " | pale and anxious. |
| In phthisis.................... | " | " | pale and thin. |
| In malignant disease.......... | " | " | sallow and thin. |
| In icterus..................... | " | " | yellow and thin. |
| In renal disease............... | " | " | thin, puffy, and anæmic. |
| In peritonitis................. | " | " | anxious and dragged. |
| In uterine disease............. | " | " | sallow and haggard. |

Marshall Hall* thus describes a countenance which he considers typical of the acute form of dyspepsia: "This affection is

* "On Diagnosis" London, 1817.

accompanied by some paleness or sallowness, and a dark hue about the eye. The lips are slightly pale and livid. The cutaneous vessels exude a little oily perspiration, and the muscles of the face, and especially of the chin and lips, are affected with a degree of tremor, particularly on any hurry or surprise, or on speaking."

THE NOSE.—The nostrils are of some practical interest from a medical point of view. They *dilate* forcibly and rapidly in difficult respiration, when produced by disease;* and *itching* of the nostril is regarded by many authors as a valuable diagnostic sign of intestinal worms.† The nose seldom points directly forward, being, as a rule, slightly inclined toward the right side. This fact is explained by Béclard as the result of the habit of wiping the nose with the right hand, since, in left-handed people, the opposite deflection exists. The nose of a face perfect in its outline should be one-third of the length of the distance from the root of the hair to the chin; but, in certain races, the variation from this rule affords a special physiognomy. The integument which covers the nose is very firmly attached to the muscles underneath it by a cellulo-fatty layer. Blandin‡ lays great stress upon this fact as explaining the infrequency of œdema of this region, and as an effort on the part of Nature to preserve the uniformity of contour of the nose, which would be seriously impaired by any local swelling of the face, were the skin over the nose loosely attached. The nose is extremely vascular; hence the custom of surgeons to replace severed portions of the organ, even if completely detached, with a hope of obtaining union. Among the ancients, amputation of the nose was practiced upon the criminal classes, and the operation of rhinoplasty was first suggested as a means of relief for those so disfigured.

The redness of the nose after an attack of crying indicates a connection between the sympathetic supply of the capillary vessels of the nose and that of the capillaries of the lachrymal apparatus; hence any form of irritation of either of these localities is liable to be accompanied by symptoms referable to

---

* Sir Charles Bell.

† Peiper.

‡ Op. cit.

the other.* Injury to the nose, resulting in fracture, often leaves a permanent facial deformity, and, even when no evidences of serious injury can be ascertained by external examination, cerebral symptoms are liable to follow, as fracture of the base of the skull may result, from a transmission of the force through the perpendicular plate of the ethmoid bone.† Vascular tumors of the region of the nose are not uncommon, while a prominence of the capillary vessels of the nose is met with in the aged as the result of a defect in the contractile power of their coats.‡

*Marked elevation of the nostril* is regarded by some authorities§ as an indicator of pain within the cavity of the thorax.

THE EYE.—" It may appear to many a superfluous task to attempt to judge of the character of an individual by a glance at his face, but, whatever may be thought of the possibility of laying down strict rules for such judgment, it is a fact of every-day occurrence that we are, almost without reflection on our part, impressed favorably or unfavorably with the temper and talents of others by the expression of their countenance. The face acquires its expression also from bodily habits and from intellectual or sensual pursuits, so that we may pass from the lofty and expanded forehead, with the small, well-formed mouth of the philosopher, down to the shallow front and protruded muzzle of the negro, whose habits are more bestial than those of the animals he chases for the support of his life."||

The intimate communications between the fifth, the seventh, and the sympathetic nerves, through the media of the ciliary, optic, and Meckel's ganglia, would lead us to expect that the eye should exhibit, in its altered appearance, the derangement of internal structures. " When a glance of this organ is caught, what a field of mute expression is open to the mind! This silent and instructive index of the whole man may be bright or dull, heavy or clear, half shut or unnaturally open, sunken or protruded, fixed or oscillating, straight or distorted, staring or

* Blandin, op. cit.

† Holden, "Human Osteology." London, 1855.

‡ Béclard.

§ Marshall Hall, op. cit.

|| Corfe, op. cit.

twinkling, fiery or lethargic, anxious or distressed; again, it may be watery or dry, of a pale blue, or its white turned to yellow."*

The pupils may be contracted or widely dilated, insensible to or intolerant of light, oscillating or otherwise, unequal in size, or changed from their natural clearness of outline. The noble arch of the brow speaks its varied language in every face of suffering humanity. It may be overhanging or corrugated, raised or depressed; while the lid of the eye, an important part of this vault, exhibits alternations of puffiness or hollowness, of smoothness or unevenness, of darkness or paleness, of sallowness or brown discoloration, of white or purple. Lines intersect this region, and the varied tints are perpetually giving new color, new feature, new expression, by their shadows. If the frontal muscle acts in connection with the corrugator supercilii, an acute deflection upward is given to the inner part of the eyebrow, very different from the general action of the muscle, and decidedly expressive of debilitating pain, or of discontent, according to the prevailing cast of the rest of the countenance. An irregularity of the pupils of the two eyes indicates, as a rule, pressure upon nerve centers or upon the optic nerve itself.† In adynamic fevers the eyes are heavy and extremely sluggish, and are, as a rule, partially covered by the drooping eyelid; while in certain forms of mania they are seldom motionless.‡ This latter peculiarity is also often noticed in idiocy.

In the so called "Bell's paralysis," due to failure of the facial nerve, the eyelids stand wide open and can not be voluntarily closed, since the orbicularis palpebrarum muscle is paralyzed. This condition may be further recognized, if unilateral, by a smoothness of the affected side, since the antagonistic muscles tend to draw the face toward the side opposite to the one in which the muscular movement is impaired; an inability to place the mouth in the position of whistling, since for this act the two sides of the face must act in unison; loss of control of saliva, which dribbles from the corner of the mouth; and a tendency to

---

* Corfe, op. cit.

† Ferrier, "The Localization of Cerebral Disease." New York: G. P. Putnam's Sons, 1879.

‡ Connelly, *Med. Times and Gaz.*, 1861–2.

accumulation of food in the cheek, since the buccinator muscle no longer acts.

FIG. 6.—"Bell's Paralysis." (Modified from Corfe.)

When the third pair of nerves are affected upon either side, the upper eyelid can not be voluntarily raised, for the levator palpebræ muscle fails to act; and the eye is caused to diverge outward, since the external rectus muscle, not being supplied by the third pair, and having no counterbalancing muscle, draws the eye from its line of parallelism with its fellow. In photophobia, attempts to open the eye create resistance on the part of the patient, since the entrance of light causes pain; while, as death approaches, or in the state of coma (save in a few exceptions), the eyes are usually open. In cardiac hypertrophy an unusual brilliancy of the eye is perceived,* since the arterial system is overfilled from the additional power of the heart. A peculiar glistening stare exists during the course of scarlet fever, which is in marked contrast with the liquid, tender and watery eye of measles.† Many diseases of the eye itself tend to greatly alter the normal expression of the face. Prominently among these may be mentioned cataract, glaucoma, cancer, staphyloma, exophthalmus, iritis, conjunctivitis, amaurosis, etc., but the special peculiarities of each need not be here described.

Abnormalities of the pupils may afford the practitioner material aid in diagnosis. The pupils are found to be dilated during attacks of dyspnœa and after excessive muscular exertion,‡ in the latter stages of anæsthesia, and in cases of poisoning from belladonna and other drugs of similar action. A contracted state of the pupils exists during alcoholic excitement, in the early stages of anæsthesia from chloroform, and in poisoning by morphia and other preparations of opium, physostigmin, chloral, and some other drugs. Paralysis of the third cranial nerve creates a dilated condition of the pupil of the same side, since that nerve controls the circular fibers of the iris.

Growths within the deeper portions of the orbit tend to create a displacement of the eye forward, and thus to cause an apparent increase of that organ in size. A similar condition may also result from abscesses or the growth of tumors within the cavity of the antrum. In the so-called Basedow's disease,§ an abnormal prominence of the eyes accompanies a simultaneous enlargement of the thyroid gland. The eyelashes, if abnormal, not only in themselves create deformity, but also, by causing irritation of the conjunctiva, produce an alteration in the normal expression of the eye.

THE CHEEK.—The cheek is capable of a great variety of move-

* Loomis, "Lectures on Diseases of the Respiratory Organs, Heart and Kidneys" New York: William Wood & Co., 1874.

† J. Duggan, quoted by Haviland Hall: "Differential Diagnosis," Philadelphia, 1879.

‡ M. Foster, "Text-Book of Physiology," 3d ed. London: Macmillan & Co., 1879.

§ F. von Niemeyer, "Text-Book of Practical Medicine." Translated by Hackley and Humphrey. New York: D. Appleton & Co., 1869.

ment. During the reception of liquid or solid food into the mouth, it is of the greatest assistance, since by its movements the two acts are greatly facilitated; during mastication, the buccinator muscle helps to force the food between the jaws, which are brought into apposition and rubbed together; and, finally, the cheek can act as an important factor in producing that peculiar type of countenance which is so strongly indicative of the desire of taking nourishment. The respiratory motions of the cheek are manifested in the acts of gaping and blowing, and in the exhibition of intense passion, in which the malar region is markedly in sympathy with a general excitation of the whole respiratory apparatus.

The cheek may become the mirror of the soul. When the feelings are gay, it is drawn outward and upward; but, when the mind is depressed or saddened, it is drawn obliquely downward. If these movements be carefully noted, it will be perceived that the movable point of the cheek is situated in the immediate vicinity of the naso-labial groove; * since the attachments of several of the small fascial muscles at about this point tend to draw the anterior part of the cheek outward from the line of this groove. It may be noticed, as a matter of interest, that when the mental impressions are slight and trivial, no traces of their effect upon the face are left upon the cheek; but, when they are of a serious or prolonged character, deep and permanent grooves are formed, which are of interest to the physiognomist as an indication of the temperament, and to the medical adviser as often of positive value in diagnosis. In the young child, the cheek, which is at nearly the same instant alternately moistened with a tear or decked with a smile, preserves in the healthy state the roundness which marks that happy age; but in the adult, the cheek, on the contrary, presents numerous lines and wrinkles, and this appearance becomes still more apparent as old age approaches.

There are, however, lines in the cheeks of the aged which should not be mistaken for evidences either of the temper-

* Blandin, op. cit.

ament or of disease, since they are produced simply by the approximation of the jaws. Lavater,* in his work upon physiognomy, locates most of the sentiment of the face in the cheek, and draws comparisons between the base and jealous face and that which is generous and noble, as a support to his theory.

The color of the cheek varies much, both as a direct result of the passions and from special diseased conditions, which have been mentioned previously in this article. In fear and envy, the cheek is usually pale and colorless, while in love, embarrassment, or anger it is often uncommonly red. To the physiologist, these changes are a beautiful exhibition of the sympathy which exists between the mind and the circulatory and respiratory systems, which are seldom influenced except simultaneously. The changes in the cheek which affect expression, like the respiratory motions, depend chiefly upon the influence of the facial nerve; and thus it is that children and females, in whom the nervous system is generally more susceptible to impressions, also present, to the greatest degree, more or less transient modifications of the cheek. The cheek suffers a diminution in its fat as age advances, and when the teeth have been lost the approximation of the jaws forces the redundant cheek outward; and its flaccidity, from the loss of fatty tissue, throws it into folds, which are not present in the face of the infant.

The cheek approaches a triangular form in the infant, but it becomes quadrilateral when the teeth are developed; and in the old man, as the teeth are lost, it again returns to the triangular form as in infancy. The fact that the maxillary sinus is very imperfectly developed in the child, and gradually increases as age advances, explains to a great extent why the triangular form tends to become quadrilateral; and the frequency of abnormal protrusions of this region is explained by growths or the accumulation of fluid within this cavity. The changes in the cheek produced by advancing years are also illustrated in its color. In the child, the bright rose tint, which accompanies exertion and frequently the hours of sleep, bespeaks health and general activity; but in adult age this coloring tends to disappear, and in old age

* Op. cit., Hunter's edition.

the cheek often assumes a striated redness, which is due to an abnormal dilatation of the capillary vessels, especially the veins. The vascularity of the cheek renders the occurrence of erectile tumors common in this region; and the elasticity of the tissues affords an anatomical explanation of the little disfigurement which follows the removal of large portions of the cheek, in case surgical interference is demanded from any cause.

THE LIPS.—Certain deformities of the face are common in the region of the lips and mouth. Among these may be mentioned the condition of deficient closure, which is the normal condition of the hare, and to which the term "hare-lip" is applied. This deformity may be associated with that of fissure of the hard palate, and often with imperfect development of the soft palate; and thus not only is the countenance impaired, but the power of sucking, natural to the infant, is destroyed, and the articulation of words is subsequently rendered imperfect. The vascularity of the lips renders the development of erectile tumors of this region not infrequent; while hypertrophy of the tissues forming the lips may occur as one of the types of facial deformity.

The lips of the young child are very much longer in proportion to the face than those of the adult, and their increased length renders the act of sucking easier to the infant than if the teeth were present, since the lips can be made almost to cross each other and thus closely embrace the nipple. When the teeth are formed, the excessive length of the lips diminishes, and the expression of the face is thus greatly altered; while, in the old man, as the teeth are lost, the lips again become very long, which accounts for their projection forward when the mouth is closed, and which gives to those advanced in years the peculiar pouting expression so often seen.* The excessive length of the lips in the aged furthermore acts as a hindrance to mastication, and often renders the articulation of words extremely indistinct.

In sickness, if the angle of the mouth be depressed, pain and languor may be read; and, when the corrugator supercilii muscle

---

* Bandin, op. cit.

coöperates with the depressor muscles of the mouth, acute suffering is proclaimed.*

Extreme pallor of the lips is observed in excessive hæmorrhage, in purpura, in chlorosis, etc.; deep lividity denotes a defective oxygenation of the blood, and occurs chiefly in diseases of the lungs, heart, and larynx; while pale lividity occurs in cases where the circulation of the surface is languid or imperfect.† In painful affections of the abdominal organs, the upper lip is usually raised and stretched over the gums or teeth, so as to give a diagnostic expression to the countenance, which is considered by some as of great value. In anasarca of the face, the lips, eyes, and cheeks are most affected, since the subcutaneous cellular tissue in these regions admits of distention more readily than in those regions where it is not so loose.

DEFORMITIES OF THE FACE.—Among the extraordinary deformities of the orbital region, may be casually mentioned those rare cases of absence of the eyes, and the union of the two orbits, as reported by Tenon and Bartholine. The eyelids may also be found deficient or united at birth; and occasionally turned in or out, when the skin and the conjunctiva are of unequal length. The last type of deformity is most frequently the result of cicatrization of the tissues of the face, following an injury; while adhesions of the eyelids to the globe of the eye may be either a congenital defect or the result of inflammatory processes. The pupils may be absent at birth, or may be partially incomplete; ‡ while deformities of this aperture may also be acquired as the result of adhesions between the iris and the cornea or the crystalline lens, or as the result of an operation in which portions of the iris are exercised for the relief of glaucoma.

The entire absence of the face at the time of birth has been recorded by Lecart, Curtius, and Béclard; while in numerous instances the median portions of the face have been absent, or the existence of deep central fissures in the face has been detected. Cases are on record where all evidences of the existence of the nostrils are absent, termed "anarina"; those where the mouth has

---

* Corfe.

† Marshall Hall, op. cit.

‡ Blandin, op. cit.

been found absent, termed "astomia;" and those where a double nose has existed, as recorded by Béclard. In these abnormalities, as in those where the cranium has been partially or totally wanting, an arrest of the process of development at an early stage of fœtal life must have occurred, the date of which in pregnancy may be roughly estimated by the extent and situation of the deformity. In cases of senile atrophy of the forehead, the bones are sometimes completely absorbed, and hernia of the encephalon may thus spontaneously be produced.

Tumors of the face always create a deformity, which is confined to the anatomical region affected; some of which have already been referred to in this article in the treatment of certain of the special features. Many conditions of the face, which may properly be spoken of as deformities, are dependent upon disease. Some of those which affect the eye and its appendages, and others which are due to injury of nerves or to disease of nerve centers, will be described later on, among the special types of physiognomy which are of interest in their bearing upon general diagnosis. Severe types of ulceration, as it occurs in lupus and carcinoma, often create so extensive a destruction of tissue as to give rise to hideous deformities, but they have no special bearing upon the diagnosis of the existing disease.

SPECIAL TYPES OF FACE.—Many of the specific forms of disease have their special physiognomy. As examples of this fact, scrofulous children inherit either a velvety skin, dark-brown complexion, dark hair, dark brilliant eyes, and long lashes, with the lineaments of a face finely drawn and expressive; or a fair complexion, thick and swollen nose, broad chin, teeth irregular and developed late, inflammation of the Meibomian glands, scrofulous ophthalmia, eruptions of the head, nose and lips, and enlarged cervical glands.*

Hippocrates † describes a characteristic expression, which has been called from him the "facies Hippocratica," in which the eyebrows are knitted, the eyes are hollow and sunken, the nose is very sharp, the ears are cold, thin, and contracted, with

* Williams, op. cit.

† "Prognostics" (Adam's translation).

marked shriveling of the lobules; the face is pale and of a greenish, livid, or leaden hue; and the skin about the forehead is tense, dry, and hard. This type of countenance is a most frequent indicator of impending death from chronic disease, or in an acute form of disease which has been unusually prolonged.

The "facies stupida" is distinguished by a dullness of expression, which is its chief characteristic. A peculiarity exists as regards the eyes, which are extremely dull, and resemble those seen in alcoholic stupor. This type of countenance is identical with the so-called "typhoid face," since it is most frequently met with either in connection with typhoid fever or with the typhoid condition associated with some other disease.*

Another type of countenance to which attention is frequently drawn is called the "pinched countenance." It can be produced artificially by exposure to cold, and is characterized by an apparent decrease in the size of the face, with a contracted and drawn expression of the features, and pallor or livid color of the skin. It is said to exist most frequently in the course of acute peritoneal inflammation.

In the long list of diseases which tend to shut off the supply of air to the lungs more or less suddenly, and in those accidents, such as choking, strangulation, smothering, drowning, etc., where the same effect is accomplished, the symptoms of apnœa are manifested in the face by flushing and turgidity, at first, and later on, by a livid and purplish color. The veins of the neck become markedly swollen, and the eyes seem to protrude from their sockets. A loss of consciousness, and possibly convulsions, precedes death.†

The countenance of extreme anæmia is seen in those cases where from sudden or gradual hæmorrhage, the prognosis is rendered alarming. The phenomena which attend this mode of dying are pallor of the face, with a peculiar leaden or clay-like hue, ‡ cold sweats, dimness of vision, dilated pupils, a slow, weak, irregular pulse, and speedy insensibility. With these symptoms

* Finlayson, "Clinical Diagnosis." Philadelphia: H. C. Lea, 1878.

† Watson, "Practice of Physic" (Condie's edition).

‡ Sir Charles Bell, op. cit.

are frequently conjoined nausea, restlessness and tossing of the limbs, transient delirium ; a breathing which is irregular, sighing, and, at last, gasping ; and convulsions before the scene closes.

FIG. 7.—Face after Hæmorrhage. (Modified from Corfe.)

The expression of the countenance is typically marked in certain of the inflammatory diseases of the eye.* In strumous ophthalmia, the child's brow is knit and contracted, while the ala nasi and the upper lip are drawn upward. Those muscles which tend to exclude the light from the inflamed organ, without shutting out the perception of external objects, are called into action ; thus producing a peculiar and distinctive grin. In severe cases, the child will sulk all day in dark corners, or, if compelled to stay in bed, will bury the face in the pillow, since the exclusion of all light tends greatly to diminish the suffering. If brought to the window, the eyes are shaded with the hands or the arms ;

* Haynes Walton, "Operative Ophthalmic Surgery." Philadelphia, 1853.

and, if the eye be opened, a profusion of hot, scalding tears will enter the nose and give rise to sneezing, or flow over the face and cause excoriation of the adjoining parts. This special intolerance of light seems to be a chief characteristic of this type of trouble, since it is often greatly out of proportion to the redness which indicates the extent of the inflammation present. In catarrhal ophthalmia, the inflammation seems to be confined to the conjunctiva and the Meibomian follicles. The eyelids are glued together by the lashes, which are bathed in the excessive secretion of the conjunctiva or of the inflamed follicles; and a redness of the surface of the eye, with some pain and uneasiness, is the only other symptom of special diagnostic value.

The deformity of iritis is characterized by a redness of the sclerotic; a change in the color of the iris, and in its general appearance, as compared with the healthy eye; an irregularity in the pupil, produced by adhesion of the iris to the adjacent structures; possibly immobility of the pupil, as the result of such adhesions; and a visible deposit of coagulable lymph. The pupil, in acute iritis, seldom dilates in the dark, on account of the intense congestion which exists;* and it is usually smaller than that of the unaffected eye. Some pain and excessive photophobia are usually also present in attacks of acute iritis. There is something very peculiar in the expression of the countenance of a person suffering from amaurosis, by which alone the physician may almost recognize the disease. Such a patient enters a room with an air of great uncertainty as to movement; the eyes are not directed toward surrounding objects; the eyelids are wide open; and the patient seems gazing into vacancy. This unmeaning stare of the face is due, in great measure, to an absence of that harmony of movement and expression which results largely from the information obtained by the exercise of vision.† This seeming stare at nothing is not observed in patients who are blind in consequence of opacity of the crystalline lens or of its capsule, i. e., in consequence of cataract. They, on the contrary, while they cannot see, still seem to look

---

* See the experiments of Mosso, quoted by Michael Foster.

† Watson, op. cit.

about them, as if they were conscious that the power of sight remained in the retina, although the perception of objects was shut out from it. Patients, afflicted with cataract, who cannot detect the existence of a gas jet or a candle in a dark room, are not fit subjects for operation, as the existence of trouble behind the lens may safely be surmised; since the periphery of the lens seldom becomes opaque to such an extent as to prevent the perception of light by the retina, even if the outline of objects cannot be perceived.

The countenance of chronic hydrocephalus is perhaps the most typical of any of the conditions to which the attention of the physician or surgeon is directed. In it, the frontal bone is tilted forward, so that the forehead, instead of slanting a little backward, rises perpendicularly, or even juts out at its upper part, and overhangs the brow. The parietal bones bulge, above, toward the sides; the occiput is pushed backward; and the head becomes long, broad and deep, but flattened on the top. This, at least, is the most ordinary result. In some instances, however, the skull rises up in a conical form, like a sugar-loaf. Not unfrequently the whole head is irregularly deformed, the two sides being unsymmetrical. Some of these rarer varieties of form are fixed and connate; others are owing, probably, to the kind of external pressure to which the head has been subjected. While the skull may be rapidly enlarging, the bones of the face grow no faster than usual, perhaps not even so fast; and the disproportion that results gives an odd and peculiar physiognomy to the unhappy subjects of this calamity. They have not the usual round or oval face of childhood. The forehead is broad, and the outline of the features tapers toward the chin. The visage is triangular. The great disproportion in size between the head and the face is diagnostic of the disease, and would serve to distinguish the skull of the hydrocephalic child from that of a giant. In acute cerebral diseases, the countenance is either wild and excited, or lethargic and expressionless.*

Thoracic affections are all accompanied by more or less change in the color of the face; whereas the alteration in the natural

* Sir Charles Bell, op. cit.

hue of the features is so slight in abdominal diseases, that both the intellect and the complexion remain unaltered, up to the final struggle, though the pinched and dragged features express the acute sufferings of the patient. In pneumonia, the countenance is inanimate; the cheek, of a dusky hue, with a tinge of red; the eyelid droops over the globe; the brow is overhanging; the lips are dry, herpetic, and of a faint claret color; the chest is comparatively motionless, but the abdomen exhibits evidences of activity; the skin is hot; and the respiratory acts are usually about double the normal number, while the pulse is markedly accelerated. In cases where the dyspnœa is extreme, the patient, entirely regardless of what is going on about him, seems wholly occupied in respiring; is unable to lie down, and can scarcely speak; and the face becomes expressive of the greatest anxiety, while the expanded nostrils and their incessant movement indicate pulmonary distress.

In emphysema, the face is not only dusky but anæmic; the eyes are wide open, as the patient gazes at you; the dusky redness of the lips bespeaks the lack of proper oxygenation of the blood; the neck is thrown backward, and the mouth is slightly open, while the cheek is puffed out during the expiratory act; the distended nostril and the elevated brow stamp the case as one of dyspnœa; while the coldness of the skin shows that no acute inflammatory condition is present. If we see, in addition to these facial evidences of disease, the deformity of the chest which has been termed the "barrel-shaped" thorax, the shrugged shoulders, and the absence of that expansive movement so well marked in normal respiration, auscultation and percussion can hardly make the diagnosis more positive.

There are certain facial conditions, which so clearly tell, to the student of physiognomy, of the existence of that most prominent sign of many pulmonary and cardiac diseases, dyspnœa, that it may be well to enumerate the alterations from the normal countenance which chiefly indicate this condition. In all cases where dyspnœa is present, the brows will usually be found to be raised; the eyes will be full, staring, and clear; the nostril will be dilated, and often it may be seen to move with each respiratory act;* the mouth will commonly stand partly open, while its angles will be drawn outward and upward; the upper lip will be elevated, so as to show the margins of the teeth; and the utterance of the patient will be monosyllabic, as the rapidity of breathing renders the utterance of long sentences a matter of extreme

FIG. 8.—Countenance of Emphysema. (Modified from Corfe.)

difficulty. When we add to these symptoms those of imperfect oxygenation of blood, as is met with in all conditions where the free entrance of air is in any way interfered with, we can better understand how the clear eye becomes stupid, as coma approaches, from the carbonic-acid poisoning, and the face cyanotic from the venous tinge of the blood. It thus becomes possible for the stu-

* Lavater, op. cit.; Sir Charles Bell, "Anatomy of Expression."

dent to picture to himself the countenance which must exist in such conditions as acute laryngitis, spasmodic and true croup, thoracic tumors which cause pressure upon the lungs or the trachea, and the various conditions of the lung itself, which impede the entrance of air to the organ, but which are not of inflammatory origin, and which have, for that reason, no distinctive physiognomy.

FIG. 9.—Cardiac Dyspnœa. (Modified from Corfe.)

In cases where renal dropsy has stamped its characteristic marks upon the countenance, we may perceive the signs of dyspnœa, due to the accompanying œdema of the lungs, in the corrugated forehead, the raised eyebrow, the dilated and waving nostrils; the corners of the mouth will be found to be drawn downward and outward, expressive of some disease of the abdominal cavity; the eye will be full and anxious, indicative of suffering long continued and borne with patient calmness; the conjunctiva may present that pellucid and bleb-like condition, so often seen in this type of disease, and an œdema of the eyelid may greatly alter its appearance; finally, the waxy pallor of the complexion and the pasty and bloated cheeks show the profound anæmia of the patient.

Chronic diseases of the abdominal cavity are usually characterized by a languor of the eye and by an absence of that flash of alarm so peculiar to the acute forms of abdominal trouble;* and, if attended with steadily increasing danger to life, the corrugated brow and eyelid, the retraction of the cheek, the dragged and elongated nostrils, the depressed angles of the mouth, the protruded chin, and the parted lips, with the teeth firmly clinched behind them, still further proclaim the seat of the disease.†

FIG. 10—Face of a Patient with Obstruction at the Pyloric Orifice.

The pale face, stamped with the signs of anxiety and distress; the head raised upon two or three pillows, and the trunk similarly supported; the knitted brow, which bespeaks the cerebral disturbance; the nostrils, waving to and fro with each breath; and the jugulars which, as they lie exposed in

* Corfe, op. cit.

† M. Louis, quoted by Marshall Hall, op. cit.

the throat, show that the valves of the heart are acting imperfectly, by their pulsation or unusual distention; all may be

FIG. 11.—Cancer of the Abdominal Cavity. (Modified from Corfe.)

found in endocardial or pericardial inflammation, or in conditions of the heart dependent upon chronic valvular disease.*

The countenance of continued fevers is liable to receive a modification from their complication with some morbid affection of the head, the viscera of the thorax, or of the abdomen; the dejection produced by the latter of which is among the most important objects in the clinical study of these diseases.† In scurvy, the dirty ashy hue of the skin and its characteristic dryness; the blue and bleeding gums; the emaciation and the frequent indurations of the inter-muscular tissue of the cheeks;

* Corvisart, "Diseases of the Heart," Gates's translation. Boston, 1812.
† Marshall Hall, op. cit.

the sunken eyes, surrounded by a blue ring; and the livid tinge of the lips, make the diagnosis positive at once.

In Graves's, or Basedow's, disease, a peculiarity of the eye is produced, due to its partial protrusion from the orbit, probably from an increase of the intra-orbital fat, which stamps the disease beyond a possibility of error in diagnosis. In many cases, the inability to approximate the lids, and an absence of power to move the eye, on account of the paralysis of the muscles from the stretching which they have undergone, furnish evidence also of disease of that organ which enhances the facial deformity.

In Asiatic cholera, and in children during attacks of profuse diarrhœa, the eyeballs sink into the orbit, a dark ecchymosis appears in the region of the eyes, the lower eyelid forms a prominent fold in the region of its attachment to the cheek, the nose is pointed and sharp, and the lips, normally ruddy and full, become thin and sharply outlined. These changes are chiefly dependent upon a rapid emaciation, which follows the withdrawal of a large proportion of the water from the tissues.* In chronic atrophy, the entire absence of the adipose tissue in the subcutaneous structures causes the skin to become loose and corrugated; while various muscles become prominent from contraction (chiefly the frontalis, the corrugator supercilii, and the levator labii superioris).† Thus the so-called "senile face" or "Voltairean countenance" is produced, which is seldom to be mistaken in the child.‡

Among the diseases of the nervous system, there are certain types of physiognomy which are so characteristic as to be of the most positive value in diagnosis. Thus, in the attacks of epilepsy, the neck at first becomes twisted, the chin raised, and brought round by a series of jerks toward one shoulder. The features are greatly distorted. The brow is knit; the eyes are sometimes fixed and staring, at other times rolling about in the orbit, and again turned up beneath the eyelid, so that the cornea is covered and only the white sclerotic is to be seen; the mouth

* Vogel, op. cit.

† Marshal Hall, op. cit.

‡ Vogel, op. cit.

is twisted to one side and distorted ; the tongue is thrust between the teeth, and, caught by the violent closure of the jaws, is bitten, often severely ; and the foam which issues from the mouth is reddened with blood. The turgescence of the face indicates obstruction of the venous circulation ; the cheeks become purplish and livid, and the veins of the neck are visibly distended.

The expressions of the countenance which are produced by paralysis of any of the special nerves of the face have striking peculiarities which enable the skillful anatomist to easily detect the nerve affected. It is important to remember that, if paralysis of any nerve be the result of any form of external injury, a danger is presented in the form of tetanus, which should be guarded against by a quick comprehension of the existing malady and by all known precautions, applied with judgment based on the anatomical course and relations of the nerve affected. It is also well to bear in mind the fact, that any form of severe external violence about the face may, by causing a fracture of the bones through transmission of the force applied, cause injury to some special nerve whose course may lie far distant from the apparent seat of injury. It is not infrequent to find a fracture of the superior maxillary bone followed by symptoms indicative of a foreign body within the cavity of the antrum ; and symptoms of irritation of the nasal mucous membrane, or of neuralgia of some of the principal nerve trunks distributed to the face, may likewise follow such an accident. Violence to the vault of the skull may produce not only cerebral lesions and their subsequent evidences in the face and body, but also types of local paralysis,* produced by injury to some of the more important nerve trunks at their point of escape from the skull, in case the base of the skull has been injured.

"A slight tremor of the lips ; a hesitation of utterance ; a partial loss of power over the lips and tongue, which seem to have lost their grip, as it were, over the consonants ; a characteristic stillness of all the muscles of expression ; and a slight disparity in the pupils are the predominant features of the early

* Holden, op. cit.

stage of development of the general paralysis of the insane." * In those rare cases where the facial nerve of both sides is impaired, symptoms similar to those mentioned above exist, except that the tongue has its normal capabilities of movement, save in the perfect articulation of the labial consonants only, and that a complete absence of facial expression is present. An open mouth; a loss of control over the saliva, which constantly dribbles; an awkwardly moving or motionless tongue; and an indistinct articulation render the labio-glosso-laryngeal paralysis of Trousseau and Duchenne easy of detection.† In the so-called Bell's paralysis,‡ which has been described in previous pages of this article, the patient can not laugh, weep, or frown, or express any feeling or emotion with one side of the face; while the features of the other may be in full play. One half of the aspect is that of a sleeping or dead person; while the other half is alive and merry. This incongruity would be ludicrously droll, were it not so frightful and distressing.

During the fit of exacerbation, in an attack of tetanus, the aspect of the sufferer is sometimes frightful. The forehead is corrugated and the brow knit, thus expressing the most severe type of bodily suffering; the orbicularis muscle of the eye is rigid, and the eye itself staring and motionless; the nostril is widely dilated, indicating the extreme dyspnœa; the corners of the mouth are drawn back, exposing the teeth, which are firmly clinched together; and the features, as a whole, have a fixed and ghastly grin—the so-called "risus sardonicus." During such paroxysms, as in those of epilepsy, the tongue is liable to become protruded between the teeth and to be severely bitten.

In chorea, the facial muscles participate in the general eccentricity of movement. Watson§ thus describes the peculiarities of this strange affection: "The voluntary muscles are moved in that capricious and fantastic way in which we might fancy they would be moved, if some invisible mischievous being, some Puck or Robin Goodfellow, were behind the patient and prompted the

---

* W. H. Gairdner, Article on "Medical Physiognomy," in Finlayson's "Clinical Diagnosis."
† Finlayson, op. cit.
‡ Sir Charles Bell, op. cit.
§ Op. cit.

discordant gestures. With all this, the articulation is impeded; there is the same perverse interference with the muscles concerned in the utterance of the voice. By a strong figure of speech, the disorder might be called 'insanity of the muscles.'"

In catalepsy, the patient lies often with eyes open and staring, yet without expression indicative of life; more like a wax figure or a corpse than like a living subject. The features may be made to assume any expression, no matter how absurd, as the tissues have their normal pliability; and they will remain so placed until again mechanically altered. This same peculiarity is also present in the muscles of the extremities, and forms one of the distinguishing tests of the disease. The mental faculties are in abeyance, and all power of voluntary motion is lost. The sensibility of the body seems also to be lost.

The deformities of face and intellect which seem to be the result of residence in special atmospheric conditions, or of certain well-defined localities, are illustrated in that race of people found in Valais and the adjoining cantons of Switizerland, called "cretins." Many of these wretches are incapable of articulate speech; some are blind, some are deaf, and some suffer from all of these privations. They are mostly dwarfish in stature, with large heads, wide vacant features, goggle eyes, short crooked limbs, and swollen bellies. The worst of them are insensible to the decencies of Nature, and in no class of mortals is the impress of humanity so pitiably defaced. They are usually the descendants of parents afflicted with goître.

In that long list of pathological conditions in which the brain may be subjected to more or less compression of its substance, there are certain signs of positive value in diagnosis which may often assist the medical practitioner to locate the disease. Thus, in depressed fracture of the inner table of the skull, where the signs of external injury are absent; in abscess within the cranial cavity, during the course of meningeal inflammations; in apoplexy; in the development of intra-cranial tumors, etc., the eyelids will usually be closed and immovable; the pupils generally dilated or irregular, and always sluggish and less sensible to light than in health; the breathing will be slow and stertorous if coma exists; the special senses will be in abeyance; and the temperature will be either normal or increased. The evidences of a paralyzed condition of certain of the cranial nerves may also exist, and thus afford an additional means of determining the exact seat of the disease. A rigidity of certain muscles, if present, denotes some special irritation of the nerves which supply them, and it is, therefore, seldom present in cerebral softening, but frequently so in those cases where paralysis is produced by pressure upon nerve centers. In cases where the fifth cranial

FIG. 12.—Partial Paralysis of the Facial Nerve from Disease near the Pons Varolii. (Modified from Corfe.)

nerve has been impaired by pressure, injury, or disease, the prominent symptoms are a redness of the conjunctiva on the side of the face supplied by the affected nerve; insensibility of the cornea, nostril, and tongue on the same side; a dullness of hearing; a partial or complete loss of smell, sight, and occasionally of taste also in the anterior two-thirds of the lateral half of the tongue; and a diseased state of the gums, similar to that observed in scurvy.

While many typical varieties of countenance, which are of value to the diagnostician, have been omitted, since the limits of a single article have possibly been already overstepped, still it is to be hoped that the facts mentioned, although they are but fragmentary jottings, may tend to kindle among the medical profession a renewed interest in a subject which is rapidly being lost sight of, and the value of which is often ignored. It is not to be expected that sight alone can guide the medical attendant to unerring diagnosis; but that it may prove of the greatest value *as an aid*, can not, I think, be disputed. It is to be remembered, that a direct perceptive faculty, like that of touch, hearing, or smell, *grows with use*, and is capable of unlimited development. As with the musician, an instrument which at first produced discords becomes, under skillful hands, one of melody; so the enlightened and accomplished practitioner may often see at a glance what, to one unaccustomed to note facial changes or to interpret their meaning, would escape detection, unless a special effort was made to note and record systematically the peculiarities of each particular feature and anatomical region of the face, and the records afterward studied, as the mariner studies his chart before he attempts to direct his vessel through channels with which he is not perfectly familiar.

---

A NEW medical association has been organized in Deadwood, Dakotah Territory, with the following officers: President, Dr. J. J. Houghton; vice-president, Dr. O. B. Thompson; corresponding secretary, Dr. J. C. O'Neall; secretary, Dr. B. B. Kelly. The other members are Drs. D. K. Dickinson, Lead City, D. T.; J. A. J. Martin, Rockford, D. T.; Wm. H. Dyre, Galena, D. T.; Henry Hunter, Deadwood, D. T., and H. Williams, Central City, D. T.

THE next Annual Meeeting of the Illinois State Board of Health will be held at Springfield, Ill., January 12, 1881.

## Figures and Tables

**Fig. 1. f1:**
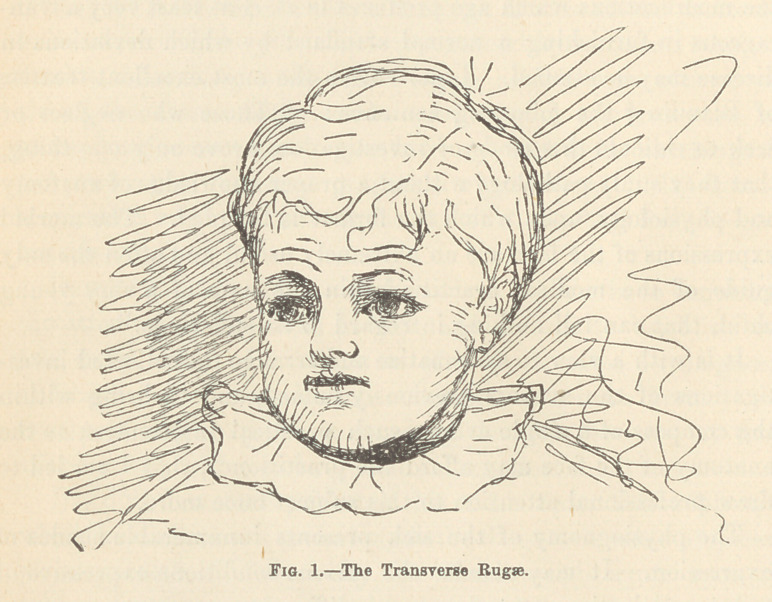


**Fig. 2. f2:**
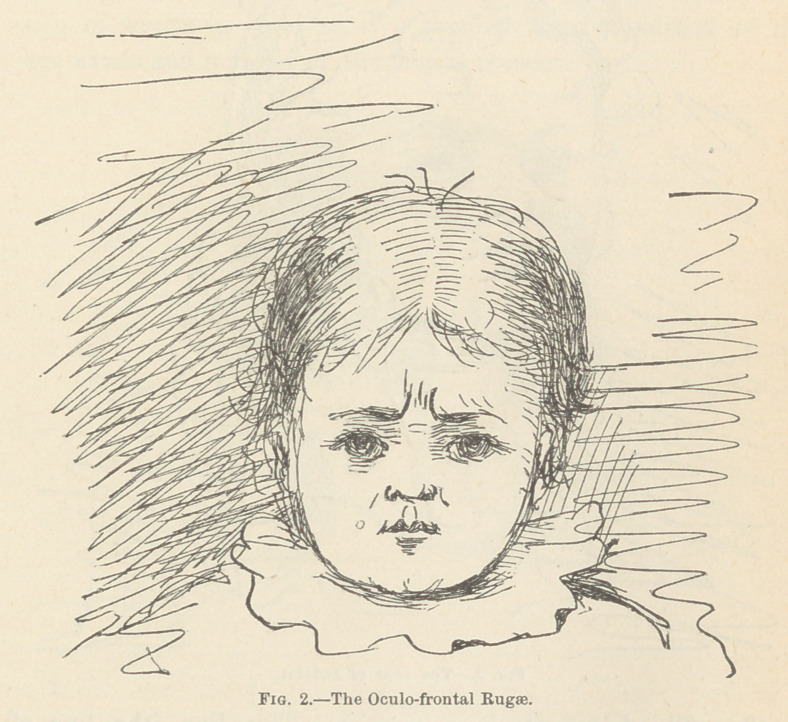


**Fig. 3. f3:**
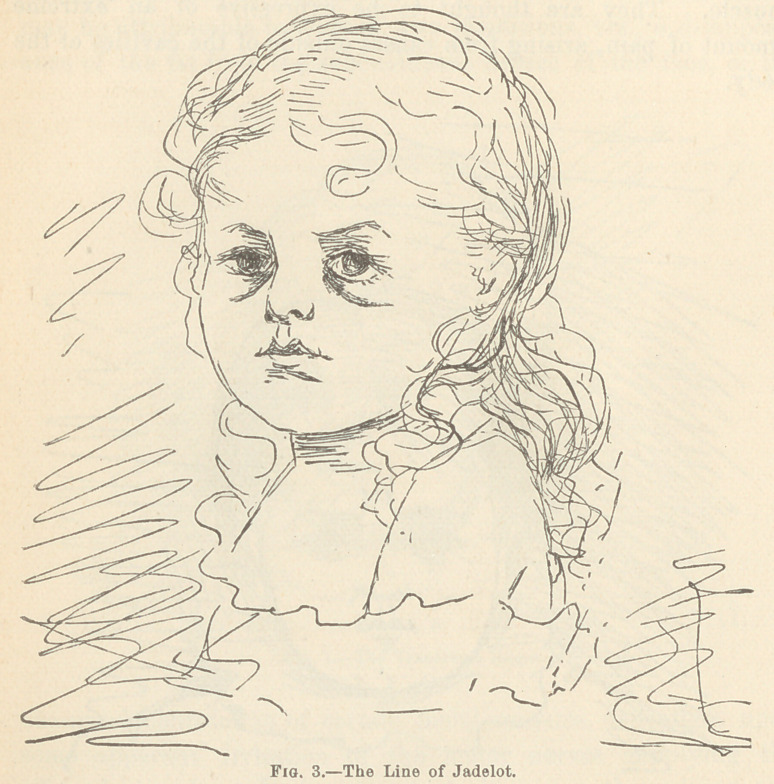


**Fig. 4. f4:**
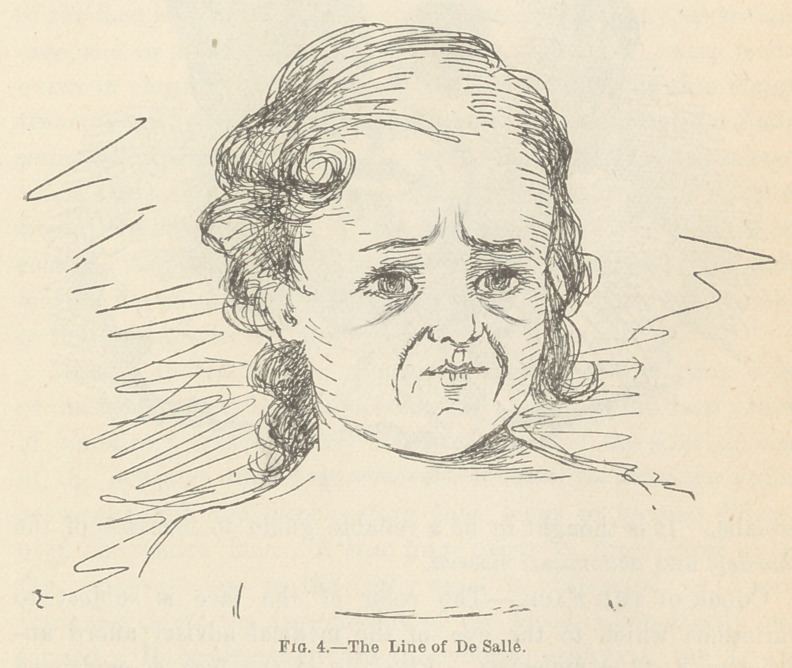


**Fig 5. f5:**
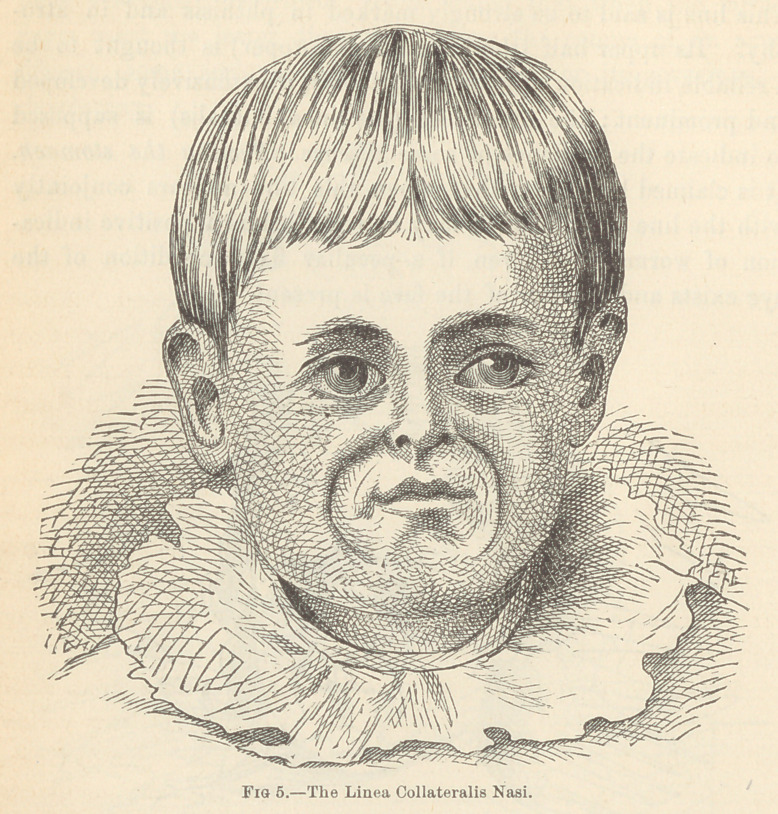


**Fig. 6. f6:**
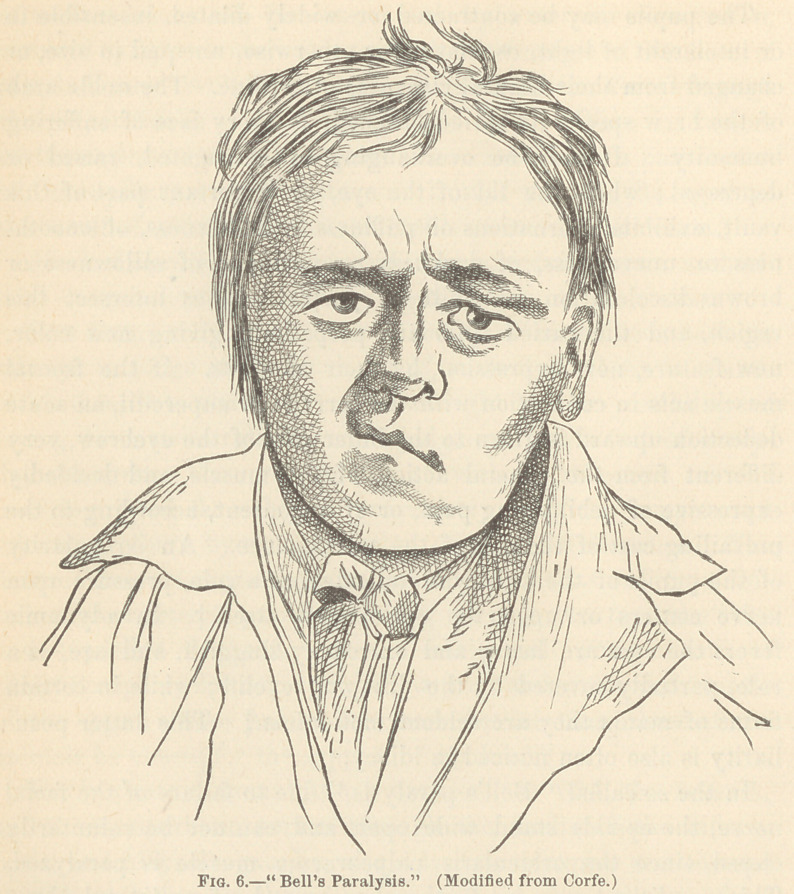


**Fig. 7. f7:**
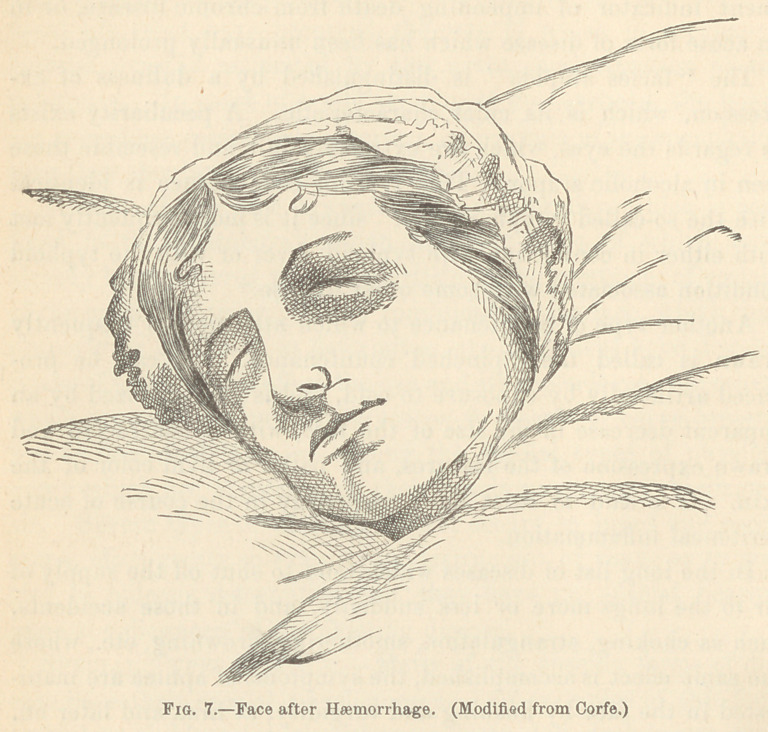


**Fig. 8. f8:**
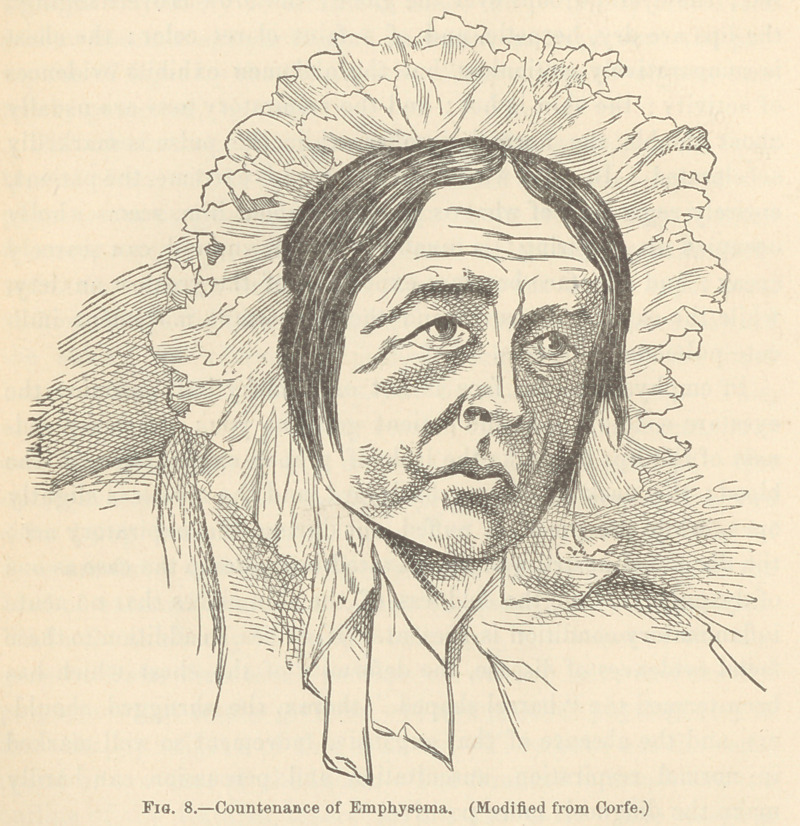


**Fig. 9. f9:**
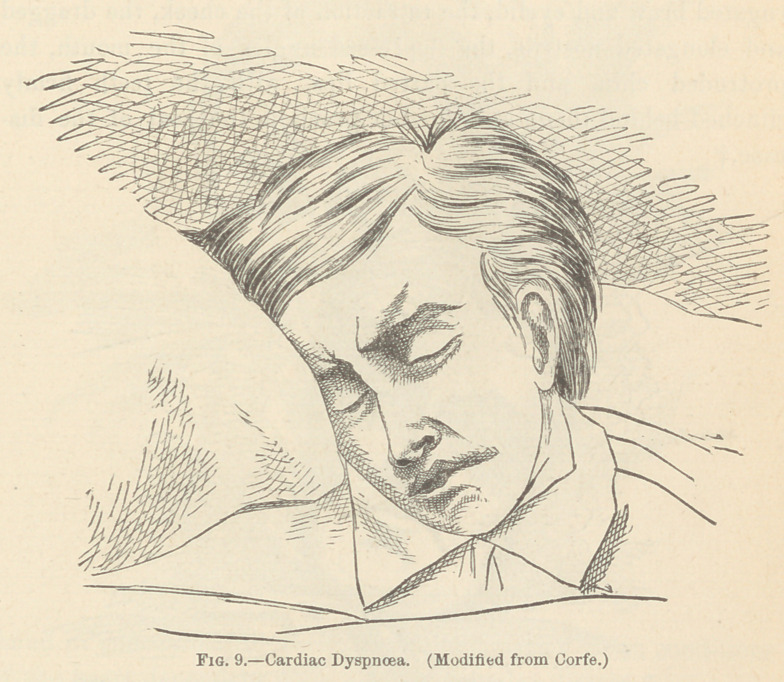


**Fig. 10 f10:**
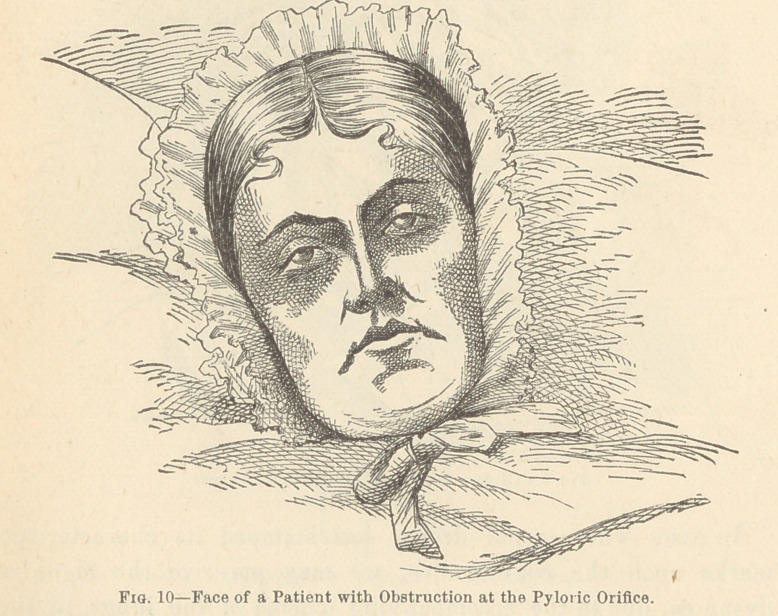


**Fig. 11. f11:**
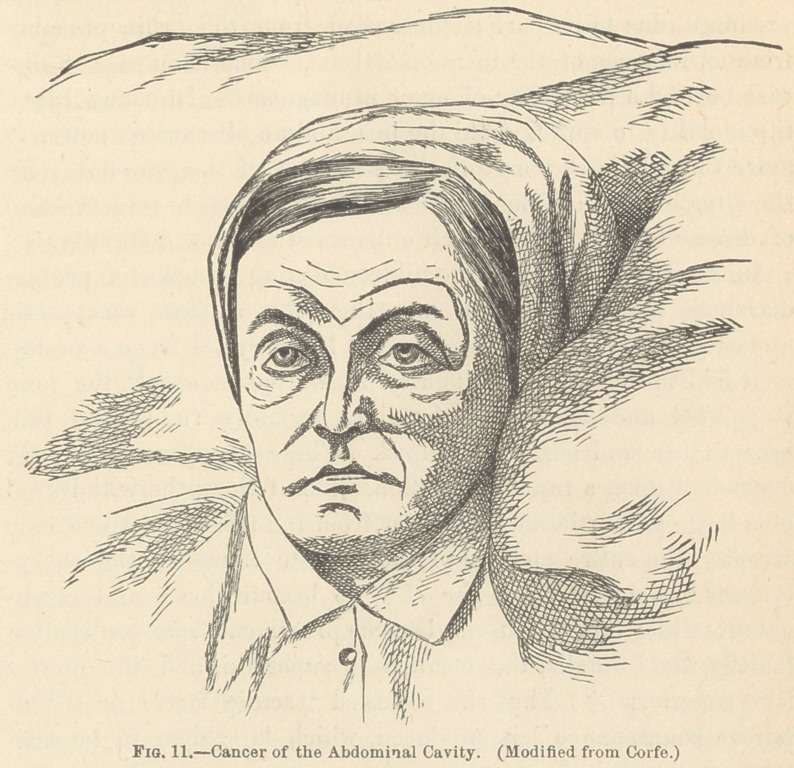


**Fig. 12. f12:**